# The Prognostic Value of Platelet Count in Patients With Hepatocellular Carcinoma: A Systematic Review and Meta-Analysis

**DOI:** 10.1097/MD.0000000000001431

**Published:** 2015-09-18

**Authors:** Qing Pang, Kai Qu, Jing-Yao Zhang, Si-Dong Song, Su-Shun Liu, Ming-Hui Tai, Hao-Chen Liu, Chang Liu

**Affiliations:** From the Department of Hepatobiliary Surgery, the First Affiliated Hospital of Medical College, Xi’an Jiaotong University, Xi’an, Shaanxi Province, China.

## Abstract

Thrombocytopenia has been acknowledged to be a crucial risk factor for cirrhosis formation and hepatocarcinogenesis in chronic liver diseases. However, to date, the association between platelet count (PLT) and the prognosis of hepatocellular carcinoma (HCC) remains inconsistent and controversial.

The aim of the present study was to determine whether PLT could be used as a useful predictor of survival in patients with HCC.

We performed systematic review in online databases, including PubMed, EmBase, and Web of Science, from inception until 2014. Studies were included if a statistical relationship was investigated between PLT and survival for HCC, and hazard ratio (HR) and 95% confidence intervals (CIs) for overall survival (OS) or recurrence-free survival (RFS) were provided. The quality of each included study was assessed by Newcastle–Ottawa scale score. To synthesize these studies, a random-effects model or a fixed-effects model was applied as appropriate. Then, we calculated heterogeneity, performed sensitivity analysis, tested publication bias, and did subgrouped and meta-regression analysis.

Finally, we identified 33 eligible articles (published from 1998 to 2014) involved 5545 patients by retrieval. A low level of preoperative PLT was found to be significantly associated with a *poor* survival of HCC. Irrespective of the therapy used, the pooled HRs for OS and RFS were 1.41 (95% CI, 1.14–1.75) and 1.44 (95% CI, 1.13–1.83), respectively. Specifically, in patients who underwent liver resection, the pooled HRs for OS and RFS were 1.67 (95% CI, 1.22–2.27) and 1.44 (95% CI, 1.04–1.99), respectively. Furthermore, patients with preoperative thrombocytopenia (PLT < 100 × 10^9^/L) had a worse OS (HR: 1.73, 95% CI, 1.29–2.32) and RFS (HR: 1.57, 95% CI, 1.31–1.87) in comparison with patients without thrombocytopenia. All our findings showed no significant changes due to the removal of any study or the use of an opposite-effects model, and there was no significant publication bias. The limitations of this meat-analysis were nonuniform cut-off values of PLT, high between-study heterogeneities, potential confounders, and a bias of publication year.

A low preoperative PLT level results in an unfavorable outcome in HCC. PLT is a simple, inexpensive, and useful predictor of survival in patients with HCC.

## INTRODUCTION

Hepatocellular carcinoma (HCC) is the fifth most common cancer and the third most common cause of cancer deaths worldwide.^1^ HCC is frequently secondary to infections with hepatitis viruses, such as hepatitis B virus (HBV) or hepatitis C virus (HCV). Because HBV infection is considered a primary risk factor for cirrhosis and HCC, 80% of HCC cases occur in areas with a high prevalence of HBV, especially in Asia-Pacific and sub-Saharan Africa regions.^2,3^ Alcohol use and obesity are also risk factors for HCC.

Despite dramatic improvements in diagnosis and treatment with improved surgical techniques and perioperative care over the past few decades, the prognosis of HCC is still poor, with an overall 5-year survival rate of approximately 5% to 6%.^4^ It is necessary for hepatologists to explore the factors affecting the outcomes of patients with HCC. Previous studies have shown that age, the presence of liver cirrhosis, a later stage, a high Child-Pugh grade, a tumor of ≥5 cm, tumor multiplicity, the presence of satellites, a high AFP level, noncurative therapy, etc.^5–9^ result in a significant decrease in the survival of HCC patients.

Platelets, the levels of which normally range from 100 to 300 × 10^9^/L in adults, are involved in the inflammatory response by releasing several cytokines, such as platelet-derived growth factors and transforming growth factor-β. Moreover, platelets are able to transport these substances to specific sites and play roles in angiogenesis, wound healing, liver regeneration, etc.^10^ Numerical and functional abnormalities of platelets result in a series of physiological/pathological changes, serious complications, and even several disorders. Most cases of HCC are accompanied by liver cirrhosis, which is primarily caused by chronic liver inflammation. Liver cirrhosis could ultimately lead to portal hypertension and hypersplenism and cause a subsequent decrease in platelet count (PLT). Furthermore, PLT,^11^ and many noninvasive indices^11–14^ that regard PLT as a critical component, have been proven to be significant diagnostic indicators for predicting liver fibrosis and cirrhosis. Studies have demonstrated that the PLT level is an independent predictor of liver-related death^15^ and HCC occurrence,^15–17^ and it plays a decisive role in the choice of therapy in HCC.^18^

However, whether the PLT level before treatment affects the long-term survival of HCC patients is controversial. We ever considered that a low PLT level results in a worse prognosis in HCC patients who received liver resection.^19^ However, several studies demonstrated that a high PLT level was associated with shorter survival or found no statistically significant association between them. Herein, we summed all relevant meta-analyses and investigated the prognostic role of PLTs in the outcome of HCC patients.

## METHODS

### Search Strategy and Selection Criteria

Two independent investigators (PQ and ZJY) performed a systematic search using the PubMed, EmBase, and ISI Web of Science databases (from inception to July 31, 2014) with no language restrictions. Our core search consisted of the terms (PLT OR platelet OR thrombocythemia OR thrombopenia OR thrombocytopenia OR “blood platelets” OR platelets) AND (prognosis OR prognostic OR survival OR mortality OR death) combined with the terms (“hepatocellular carcinoma” OR HCC OR “liver cancer” OR “hepatic carcinoma” OR “hepatic cancer” OR hepatoma). In addition, we contacted authors if full text or crucial data were not available. We retrieved the reference lists from relevant literature reviews and included the articles manually. We used EndNote X7 software to search and manage citations.

We included studies that met the following criteria: published as an original article; HCC was confirmed by pathology and (or) imaging; studied the relationship between PLTs and the survival of HCC patients, reporting hazard ratios (HRs) and 95% confidence intervals (CIs) for overall survival (OS) or recurrence-free survival (RFS) or providing sufficient data to calculate these values or the possibility of contacting authors to obtain these data; expressed the PLT level as a binary (with the lower or higher category as a reference) or continuous variable; and included a total of ≥20 patients. We excluded the following studies: studied benign liver disease or secondary liver cancer; diagnosed HCC by serum markers; had a maximal follow-up time of less than 1 year or only reported survival during hospitalization; only provided *P* values, or other conditions were present that did not permit the calculations of the effect size or 95% CI; and conference abstracts or unpublished studies. For 2 or more articles involving overlapping populations, we included the one that recruited the largest number of cases or calculated the most adjusted HR values.

### Data Abstraction

According to the selection criteria, 2 investigators (PQ and SSS) independently evaluated the retrieved studies for inclusion. Differences between the 2 investigators were estimated by a consistency check, and discrepancies between them were solved by discussion. For each included study, we abstracted the following information with a standardized data-collection protocol: the last name of the first author, publication year, region where the population resided, method of treatment, demographic data (gender and age), duration of follow-up, Child-Pugh grade, PLT cut-off value, HR value and 95% CI and confounders that had been adjusted. Meanwhile, the qualities of the studies were assessed by modified NOS (Newcastle–Ottawa scale) scores^20^ with a maximum score of 9. We defined curative therapy as liver resection, transplantation, and RFA. Other therapies, such as TACE, were classified as palliative treatments. We followed the Meta-analysis of Observational Studies in Epidemiology (MOOSE) guidelines^21^ for the present meta-analysis. All data were double-checked by 1 investigator (PQ).

### Statistical Methods of Meta-Analysis

The data from the studies considering patients with lower PLT levels as a reference group were converted to HR estimations that reflected the impact of lower PLT levels (with the higher category as a reference) on HCC. HR values for OS and RFS in all included studies, in studies involving patients who underwent partial hepatectomy, and in studies that recruited individuals who underwent RFA were merged separately. We assessed heterogeneity between studies with the Q value and I^2^ statistic value (25%, 50%, and 75% corresponded to the cut-off points for low, moderate, and high degrees of heterogeneity). A fixed-effect model was used for data showing statistically significant heterogeneity if *P* < 0.1 as determined by the Q statistic or I^2^ > 50%; otherwise, we considered that there was no obvious heterogeneity and used a random-effects model. We performed subgroup analysis and meta-regression analysis to explore the potential sources of heterogeneity. We analyzed those covariates that may have contributed to the potential heterogeneity, and at least 3 studies were included in each subgroup, for which the PLT cut-off level, treatment method, follow-up time, number of recruited patients, survival analysis (adjusted or unadjusted HR), area, and Child-Pugh grade were assessed. In addition, we performed influence analyses to evaluate whether the results could be markedly affected by a single study. We compared the calculated summary effect sizes using fixed-effects and random-effects models. Finally, publication bias was examined using funnel plots with Begg and Egger test.

We used STATA 12.0 software to analyze the data. A *bilateral P*-value of less than 0.05 was considered indicative of a *statistically significant* difference.

## RESULTS

**FIGURE 1:**
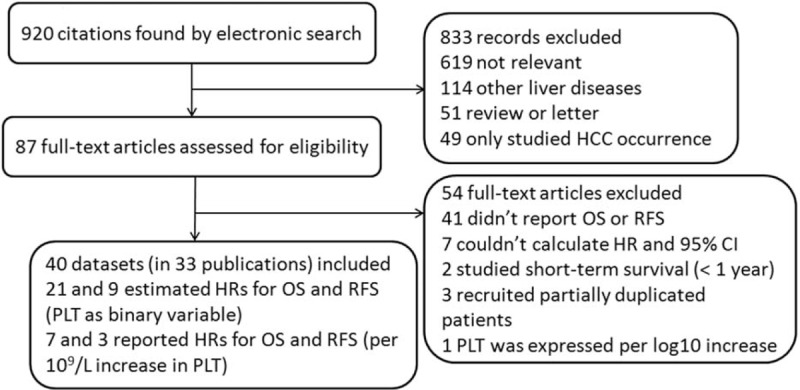
Flow diagram of search strategy and study selection.

The flow diagram of our literature search and selection process is shown in Figure 1. Of the 920 total citations, we identified 33 publications which were published from 1998 to 2014. Agreement between observers with regard to which studies to include was good (kappa = 0.937). No additional articles were included from the reference review. All studies used in our meta-analysis were published in English except for one that was published in Korean.^22^

### Characteristics of Included Studies that Expressed PLT as Binary Variable

The baselines of our included studies are summarized in Table 1. In general, this meta-analysis involved 5545 patients (4250 men and 1295 women) with a mean or median follow-up of 5 to 46 months. The PLT cut-off values ranged from 75 to 150 (×10^9^/L), which were near the cut-off point for thrombopenia (100 × 10^9^/L). The qualities of the studies were moderate to high (the range of NOS scores was 4–9, with a mean of 6.52). Four studies were performed in western regions, and the others were conducted in eastern countries. Twenty-one and 9 studies reported HR values for OS and RFS, respectively. Ten studies estimated the influence of PLTs on the prognosis of patients who underwent liver resection, and 5 studies involved patients with RFA.

**TABLE 1:**
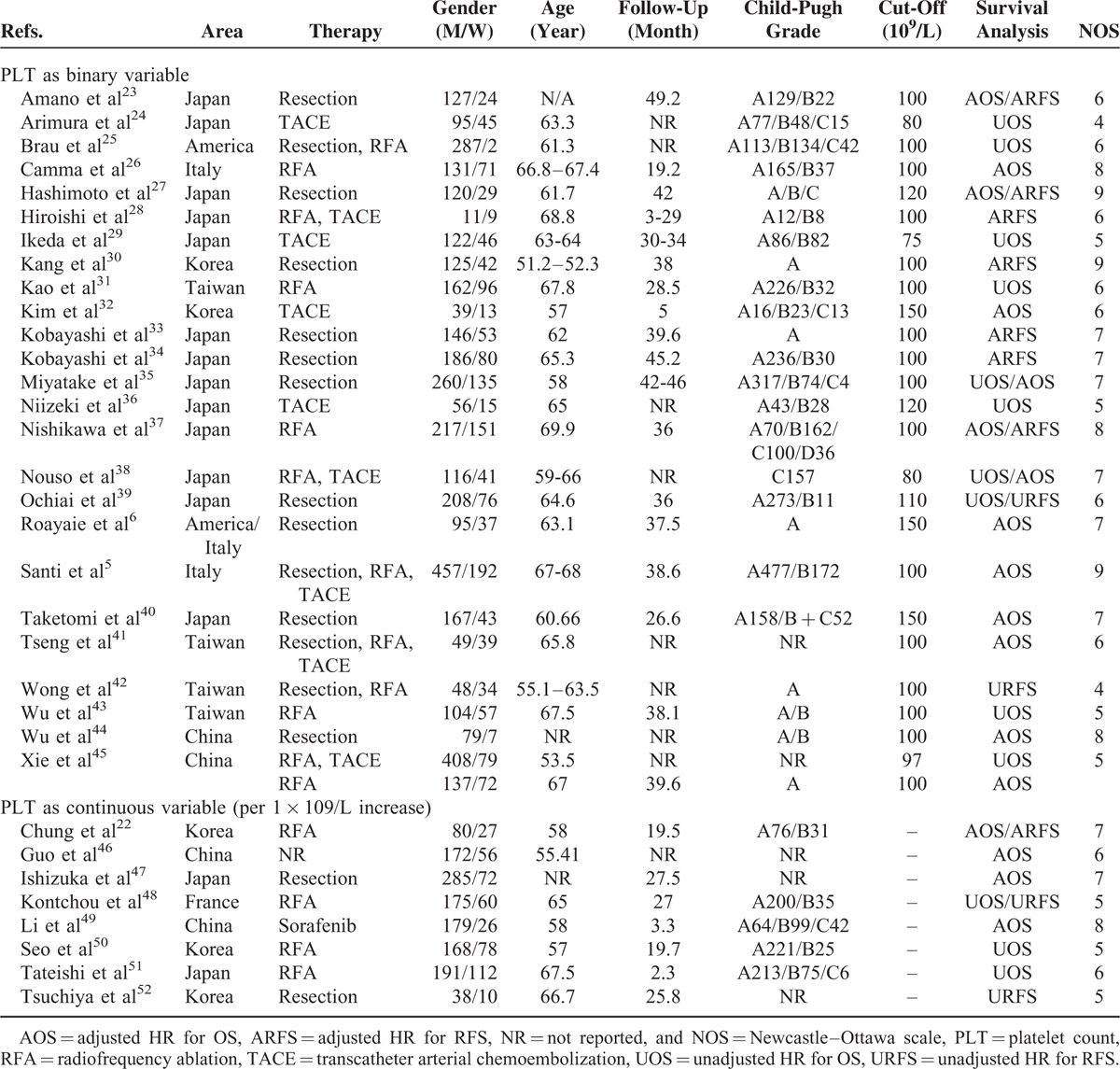
Baseline Characteristics for Studies Included in Meta-Analysis

### Pooled HR Value for All Studies

**FIGURE 2:**
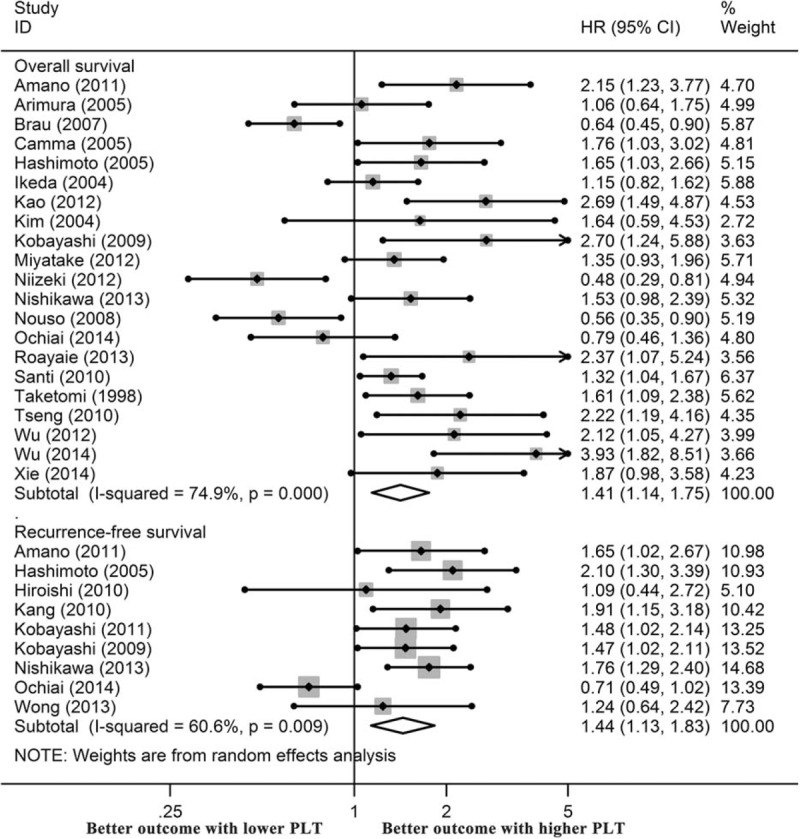
Effects of platelet count on the survival of all HCC patients.

After calculating the total effect size using a random-effects model, we found that a low PLT level before treatment indicated a poor prognosis. The forest plot is shown in Figure 2, and the pooled estimator was stratified by survival (OS and RFS). The 21 studies that analyzed OS had a pooled HR value of 1.41 (95% CI, 1.14–1.75), with a moderate degree of between-study heterogeneity (I^2^ = 74.9%, *P* = <0.001). The pooled HR value for RFS was 1.44 (95% CI, 1.13–1.83, n = 9 studies), and this value also showed a moderate degree of heterogeneity (I^2^ = 60.6%, *P* = 0.009).

### Pooled HR Values for Patients Who Underwent Liver Resection or RFA

**FIGURE 3:**
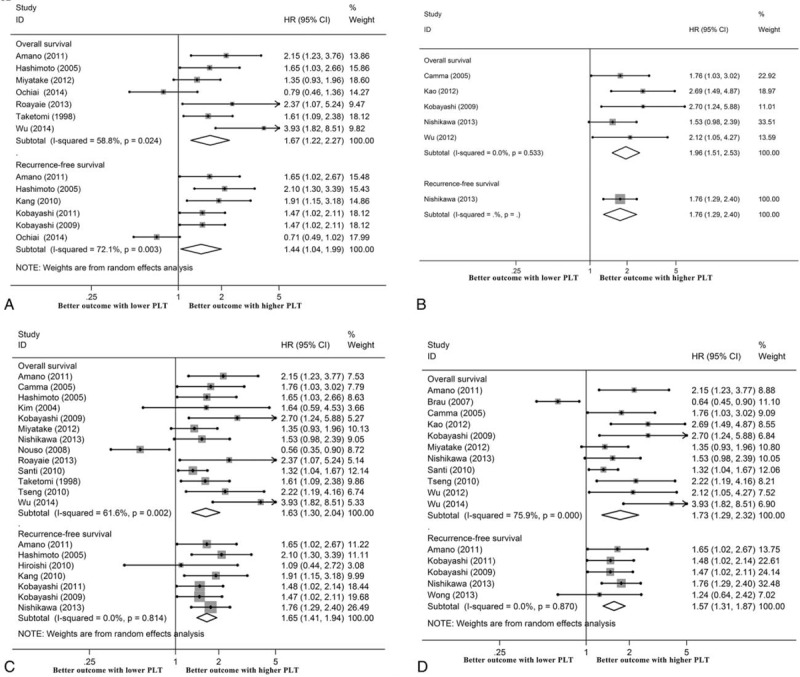
Effects of platelet count on the survival of HCC patients who underwent liver resection (A) and RFA (B), the independent effects of platelet count on the survival of HCC patients (C), and the effects of thrombocytopenia on the survival of HCC patients (D).

Next, we explored the impact of PLTs in patients who received partial hepatectomy. The forest plot is presented in Figure 3A, and the results were also stratified by survival. We demonstrated that the merged HR value for OS was 1.67 (95% CI, 1.22–2.27, I^2^ = 58.8%, *P* = 0.024, n = 7 studies). For RFS, the HR value was 1.44 (95% CI, 1.04–1.99, I^2^ = 72.1%, *P* = 0.003, n = 6 studies). Both values showed significant heterogeneity between studies; thus, a random-effects model was used. For patients who underwent RFA, 1 study provided a risk estimate for RFS, and 5 studies estimated OS rates. As shown in Figure 3B, the influence of low PLT on the OS of these patients was great, with a pooled HR value of 1.96 (95% CI, 1.51–2.53, I^2^ = 0%, *P* = 0.533).

### Independent Significance of PLTs in HCC

To explore the independent role of PLTs in the prognosis of patients with HCC, we further analyzed the studies that provided an adjusted (entered into Cox multivariate analysis) HR value for survival. By summarizing the 13 relevant studies, we found that a low preoperative PLT was an independent indictor of a poor OS (HR: 1.63, 95% CI, 1.30–2.04). In contrast, the estimated HR value for RFS was 1.65 (95% CI, 1.41–1.94) (Figure 3C).

### Effects of Thrombocytopenia (PLTs < 100 × 10^9^/L) on Survival of HCC Patients

Thrombocytopenia is a crucial predictor of cirrhosis and HCC. For 12 studies that used a PLT cut-off value of 100 × 10^9^/L, we estimated the significance of thrombocytopenia on the survival of HCC patients. As expected, patients with preoperative thrombocytopenia had a worse OS (HR: 1.73, 95% CI, 1.29–2.32) and RFS (HR: 1.57, 95% CI, 1.31–1.87) in comparison with those without thrombocytopenia (Figure 3D).

### Exploration of Risk Estimation for Per-Unit (1 × 10^9^/L) Increase in PLTs

**FIGURE 4:**
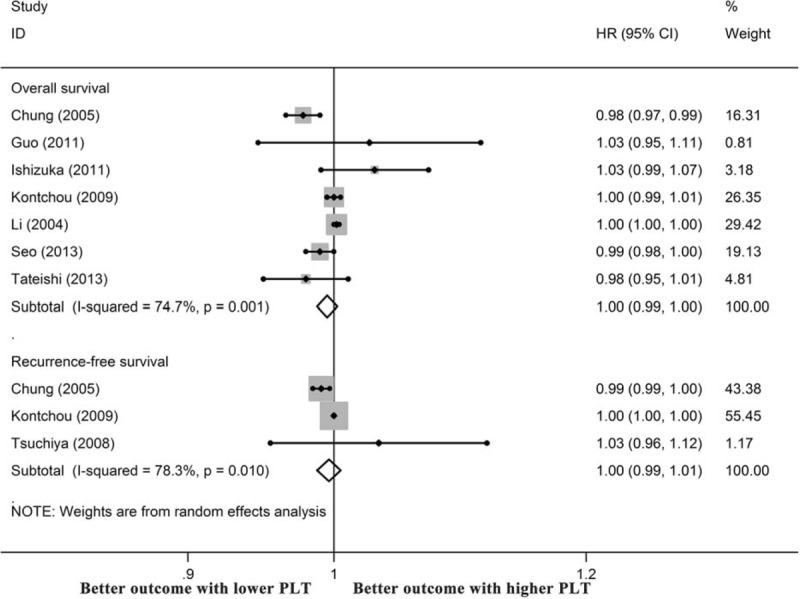
Prognostic significance of platelet count in HCC when it was expressed as a continuous variable.

Nine publications presented the HR as a per-unit increase in PLTs, and 1 of them was excluded as duplicated data. In summary, 1288 men and 441 women were recruited for meta-analysis with a median NOS score of 6 (ranging from 5 to 8) (Table 1). The pooled HR values calculated using a random-effects model showed that the PLT level was not statistically associated with OS or RFS when it was expressed as a continuous variable (Figure 4).

### Exploration of Heterogeneity

From the above analyses, we found that the pooled HR value for OS (in all studies with the PLT level as a binary variable) produced the highest heterogeneity with the largest number of studies (21 studies). To explore the source of this heterogeneity, we performed subgroup analysis and meta-regression analysis. The covariates analyzed included the PLT cut-off value (100, >100, or <100), treatment method (curative vs. palliative), follow-up time (≥3 years vs. <3 years), whether an adjustment for confounders was performed (adjusted vs. unadjusted), the number of included patients (≥200 vs. <200), Child-Pugh grade (studies with more patients with grade A than with B + C (A > B + C) vs. A < B + C or more than 80% of patients with A vs. A < 80%), and area (west vs. east). The results of our analysis are shown in Table 2. We suggested that different PLT cut-off values, treatment methods, follow-up times, and Child-Pugh grades might have affected the pooled effect size (all *P* < 0.05 for the Q statistic of between-group analysis and/or *P* < 0.05 for univariate meta-regression). The PLT level significantly influenced the prognosis of the patients treated with curative therapy, with an HR value of 1.78 (95% CI, 1.44–2.19). However, for the patients who received palliative treatment, no statistically significant association was found between the PLT level and survival. For the 3 covariates showing a *P* < 0.10 in univariate meta-regression, no significant between-group differences (all *P* > 0.05) were found as shown by multivariate meta-regression.

**TABLE 2:**
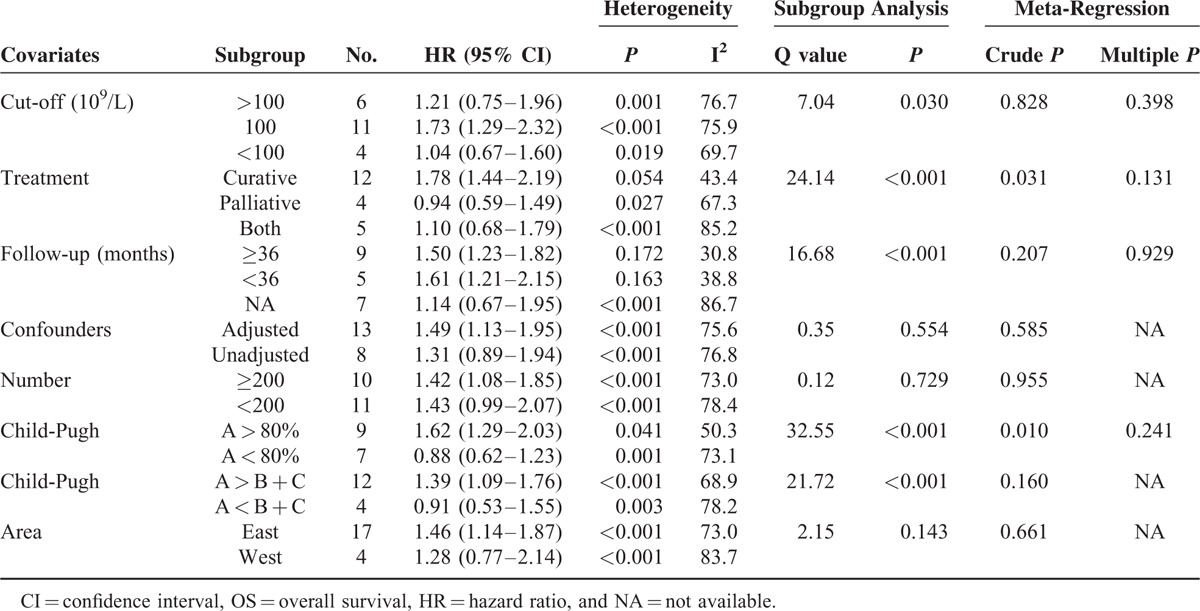
Subgroup Analysis (by Random-Effects Model) and Meta-Regression Analysis for OS Studies

### Sensitivity Analysis and Test of Publication Bias

**FIGURE 5:**
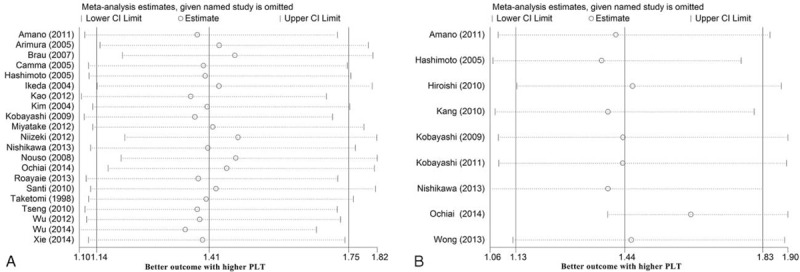
Influence analyses of the overall survival (A) and recurrence-free survival (B) studies in meta-analysis.

Sensitivity analysis was conducted to assess the consistency of the above results. We compared the use of the random- and fixed-effects models for analysis of the studies and found that there were no obvious differences between them (Table 3). Then, impact analysis was carried out for both the OS and RFS studies, and the results suggested that no single study affected the pooled estimates (Figure 5).

**TABLE 3:**
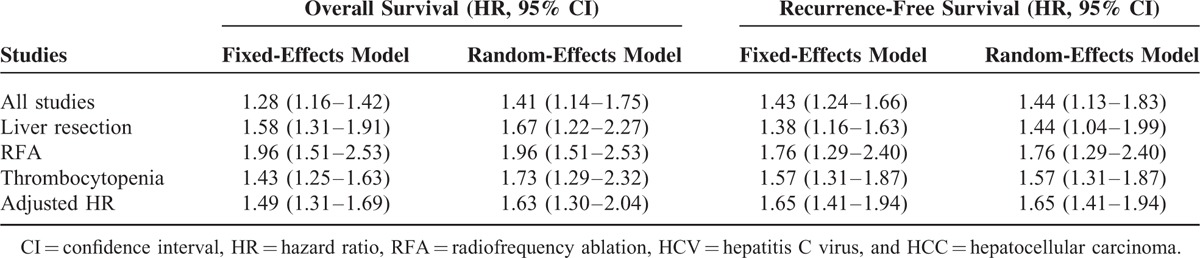
Effects of Platelet Count in Different Studies Using the 2-Effects Model

**FIGURE 6:**
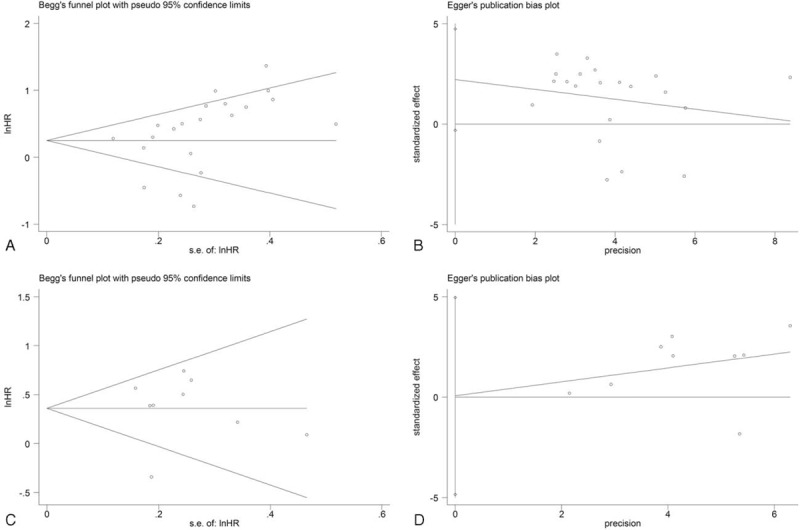
Begg funnel plot for the overall survival (A) and recurrence-free survival (C) studies, and Egger funnel plot for the overall survival (B) and recurrence-free survival (D) studies.

Finally, we constructed a funnel plot to detect the existence of publication bias, and the results indicated basic symmetry (Figure 6). We found no significant evidence of publication bias for either OS or RFS, with *P* values for OS and RFS of 0.12 and 0.75, as shown by Begg test, and 0.40 and 0.47, as shown by Egger test, respectively.

## DISCUSSION

The PLT level has been proven to be associated with the prognosis of various solid tumors.^53^ However, its effect on the survival of patients with HCC is unknown. This study provides the first estimate of the prognostic role of the PLT level, in which all relevant meta-analyses were quantitatively summarized. Our meta-analysis showed that an overall lower PLT level before treatment increased the overall risk and the tumor-free risk of death by 41% and 44%, respectively. In patients who underwent hepatic resection, the presence of a lower PLT level caused a 0.67-fold increase in the risk of overall mortality and a 0.44-fold increase in the risk of disease-free death in comparison with a higher level. A reduced PLT level was found to greatly affect the subjects treated with RFA, nearly doubling the risk of overall death. Furthermore, it was independently associated with OS and RFS in the HCC patients. Thus, patients with a low PLT level before treatment had a significantly shorter survival time after surgery (or other therapy) compared with those with a high level. Our results provide important information for the guidance of clinical management decisions and outcome prediction in patients with HCC. No significant difference was found between the results obtained using the random- and fixed-effects models, and the pooled HR value was markedly unaffected by any single study. These results indicate the robustness of our data. By subgroup analysis and meta-regression analysis, we demonstrated that the treatment method, follow-up duration, and Child-Pugh grade might be potential sources of heterogeneity.

There were some limitations and shortcomings in our study. First, the cut-off values for PLT in our included studies were not unified and greatly varied, ranging from 75 to 150 (×10^9^/L). Thus, our conclusions fail to illuminate the effect of thrombocytopenia or thrombocytosis in HCC. However, by subgroup analysis, we found that thrombocytopenia significantly aggravated the prognosis of HCC, with a 61% increase in mortality. Second, gender, age, Child-Pugh grade, and other parameters are also important prognostic factors for HCC. Whether the effect of the PLT level is independent of these confounders is still unknown because many of our included studies failed to control for them. Third, pathological examination is universally considered to be the best tool for identifying HCC. However, to obtain more data, we did not exclude those studies that assessed HCC by imaging. Fourth, the PLT level was not a useful tool when continuous variables were used. The limited amount of included data might be part of the reason for this result, and more studies are needed to validate our findings. Fifth, in our subgroup analysis and meta-regression analysis (Table 2), although studies that used a PLT cut-off value of 100 showed higher HR values than those that adopted a cut-off value of >100, the use of a PLT cut-off value of <100 was associated with the lowest HR value in the least number of studies. This inconsistence was mainly due to the limited data, the different included studies and populations, various treatment methods, various time of follow-up, as well as various Child-Pugh stages in each subgroup. All of these confounders have been identified as potential sources of heterogeneity and thus could lead to conflicting effect sizes in the 3 subgroups. Finally, although we searched databases from their available dates of inception, the studies we eventually included all published in or after 1998. As a consequence, it was almost inevitable that there would be a bias of publication year in this meta-analysis.

What roles does PLT play in the occurrence, development, and outcome of HCC and HCC-related diseases? It has been suggested that alterations in the PLT level occur throughout the entire process of HCC development, from the original inflammation and subsequent liver cirrhosis to the ultimate formation of cancer. Due to portal hypertension and some other factors, such as a decrease in thrombopoietin production in the liver^54^ and the capture of platelets by the liver,^10^ the PLT level is generally low during each stage in HCC-related diseases. The assessment of the PLT has profound significance for guiding the early diagnosis, estimating the prognosis, and directing the treatments of patients with various liver diseases. Nozaki and coworkers^55^ have performed *in vitro* and *in vivo* studies and have proven that thrombopoietin promotes liver regeneration and improves liver cirrhosis by increasing the PLT level, indirectly implying that a decrease in this level would result in the poor prognosis of patients with liver disease.

In addition to its influence on long-term survival, a decrease in the PLT level could increase the hospital mortality as well as short-term mortality of HCC patients.^56,57^ The underlying molecular mechanism is still unknown. Based on some clinical studies, we found that a low PLT level increased the risk of the occurrence of some complications and the risk of HCC recurrence, both in patients who received liver resection and in those who underwent RFA.^51,58–62^ Kubo et al^63^ have recruited 202 patients who received hepatectomy for HCV-related HCC, suggesting that only the PLT level is an independent predictor of multicentric HCC, and that it is significantly associated with the severities of active hepatitis and hepatic fibrosis (both *P* < 0.05). A previous study has also indicated that the PLT level is a predictor of portal vein invasion.^64^ In addition, a decreased PLT level has been significantly associated with an elevated AFP level^54,65,66^ and abnormal liver function.^6^ These findings may aid in the elucidation of the potential mechanism underlying the influence of the PLT level on HCC patient outcome, but more studies are needed to determine the exact mechanism.

In addition to the PLT level, several platelet-based models have been validated as useful predictors of HCC. Shen et al^67^ and Hung et al^68^ have reported that a higher aspartate aminotransferase/platelet ratio index (APRI) is significantly associated with worse survival in HCC, which is consistent with our findings. Kinoshita et al^69^ have found that an elevated platelet-to-lymphocyte ratio (PLR) is related to poor OS in HCC, which seems contradict our results. However, in contrast with the data reported by Kinoshita et al, Pinato et al^70^ have noted that patients with a PLR of >300 have a median survival time of 22 months compared to 8.0 months in patients whose PLR is <300. Thus, to date, the prognostic significance of PLR in HCC remains uncertain and controversial.

The activation of platelets is determined not only by the PLT level but also by mean platelet volume (MPV). Alterations in the MPV may be involved in some pathological process of liver diseases. Cho et al^71^ have found that the mean MPV level is significantly different between patients with HBV/HCC and controls. Subsequently, this group has indicated that the MPV/PLT ratio demonstrates a superior diagnostic capability for HCC compared with MPV alone.^72^ Previous studies have also found that MPV is an independent predictor of the severities of liver fibrosis and liver inflammation.^73,74^ However, studies reporting the association between MPV and HCC patient outcome are scanty.

In conclusion, there is a close relationship between the PLT level and survival in patients with HCC. However, to determine the influence of platelet activity (including the PLT level and MPV) on HCC and the underlying mechanism, more experimental and clinical studies are needed.
